# Atypical presentation of a methicillin-resistant *Staphylococcus aureus* brain abscess

**DOI:** 10.1016/j.idcr.2021.e01335

**Published:** 2021-11-16

**Authors:** Lauren Shute, Ken Kasper, Andrew Walkty, Stephen Ying, Sherry Krawitz, John M. Embil

**Affiliations:** a Department of Medical Microbiology and Infectious Diseases, Max Rady College of Medicine, University of Manitoba, Winnipeg, Manitoba, Canada; b Department of Medicine, Section of Infectious Diseases, Max Rady College of Medicine, University of Manitoba, Winnipeg, Manitoba, Canada; c Department of Radiology, Max Rady College of Medicine, University of Manitoba, Winnipeg, Manitoba, Canada; d Department of Pathology, Max Rady College of Medicine, University of Manitoba, Winnipeg, Manitoba, Canada

**Keywords:** *Staphylococcus aureus*, MRSA, Infection, Brain abscess

A 63-year-old female presented to health centre in northern Manitoba (Canada) with a three month history of waxing and waning headaches, worse on the right side than the left. The headaches were being managed with acetaminophen and the patient felt otherwise well. Her past medical history was significant for hypertension and diabetes mellitus. She had a left cheek abscess caused by methicillin-resistant *Staphylococcus aureus* (MRSA) six months prior to presentation that was treated with a seven-day course of trimethoprim/sulfamethoxazole (TMP-SMX). She subsequently had MRSA recovered on urine culture at four months and three months prior to presentation, respectively. Each episode of bacteriuria was treated with a three-day course of TMP-SMX.

The patient underwent a computed tomography (CT) scan of the brain which demonstrated a 2.3 × 1.0 cm lesion within the right temporal lobe with surrounding edema and possible leptomeningeal involvement, raising concern for a neoplastic process. She was referred to the nearest tertiary care hospital for further evaluation. A magnetic resonance imaging (MRI) scan demonstrated a solid right temporal lobe and meningeal lesion with enhancement but no restricted diffusion, in keeping with a suspected neoplastic process (Fig. 1A, B). The patient underwent surgical excision of the temporal lesion and tissue specimens were sent for histopathology and routine culture. Intraoperatively, the lesion appeared dense and fibrous, with no purulent material. Surprisingly, the aerobic tissue cultures yielded MRSA. Histopathology demonstrated gram-positive cocci in clusters with surrounding neutrophils and a discrete collagenous capsule, consistent with an abscess (Fig. 2A, B, C). Blood cultures obtained prior to the initiation of antimicrobial therapy were sterile. No vegetations were seen on an echocardiogram.

**Fig. 1:**
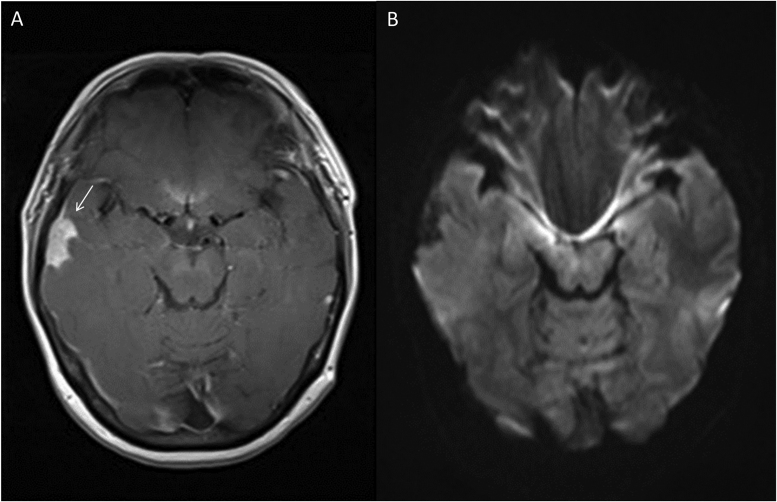
(A) Axial T1 magnetic resonance imaging scan of the brain post gadolinium demonstrating a solid right temporal lesion with enhancement (indicated with a white arrow). (B) Diffusion weighted magnetic resonance imaging scan of the axial brain with no evidence of restricted diffusion.

**Fig. 2:**
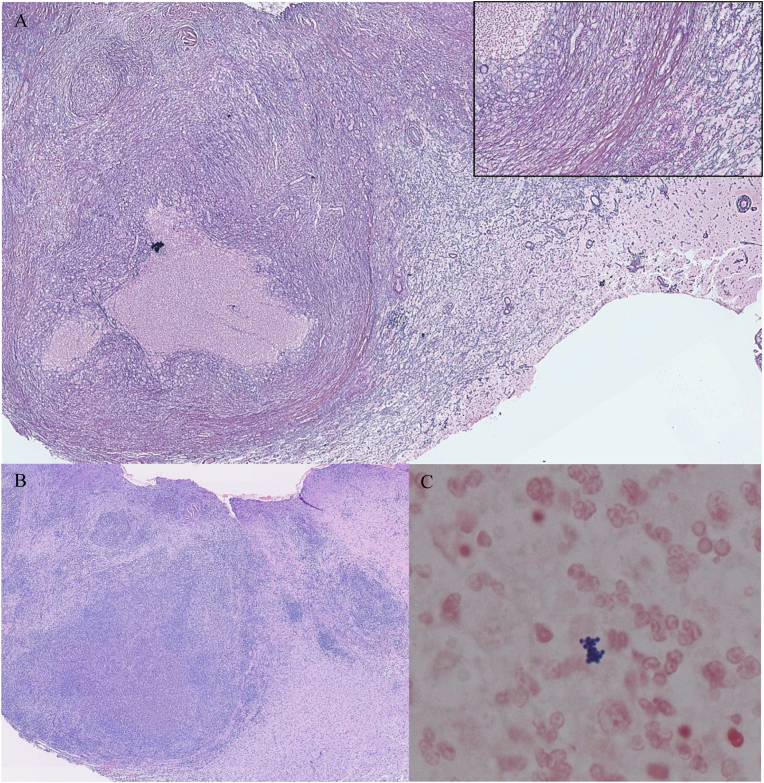
Brain abscess. (A) The inflamed granulation tissue of the abscess wall is thickened by collagen deposition and reticulin fibres. Reticulin stain, original magnification x 2. Inset: Reticulin-rich capsule of abscess. Reticulin fibres are absent at the purulent core of the abscess (left) and reticulin is attenuated in surrounding brain tissue (right). Reticulin stain, original magnification x 20. (B) Pus at the abscess core is surrounded by a thick capsule and more peripherally by brain tissue with reactive changes. Hematoxylin & eosin stain, original magnification x 2. (C) Pus at the abscess core contains neutrophils, clusters of gram-positive cocci (arrow), and necrotic debris. Hucker-Conn Gram stain, original magnification x 100.

*S. aureus* is a relatively common cause of pyogenic brain abscesses [1], [2]. Brain abscesses secondary to *S. aureus* may occur as a result of a neurosurgical procedure, penetrating cranial trauma, infection at a contiguous focus (e.g., sinusitis, facial or scalp infection), or hematogenous spread from a distant site (e.g., endocarditis) [3]. We speculate the source of infection in our patient was the preceding cutaneous abscess, which may have served as a focus of bacteremia with seeding of the right temporal lobe. The presence of MRSA bacteriuria in our case is supportive of a prior bacteremic episode. *S. aureus* bacteremia has been documented in 8–21% of patients with *S. aureus* bacteriuria [4].

The MRI findings in our case were non-specific. Diffusion-weighted magnetic resonance imaging (DWI) is usually capable of distinguishing a brain abscess from other ring-enhancing central nervous system lesions. On DWI, abscesses are typically hyperintense, indicating restricted diffusion, which is characteristic of viscous materials such as pus [5]. In the case described, the lesion lacked restricted diffusion, and was more suggestive of a neoplasm.

The case presented here is notable for the mild symptoms experienced by the patient and the neuroimaging findings which were uncharacteristic for a brain abscess. It highlights the importance of a tissue biopsy in establishing a diagnosis when uncertainty remains after a thorough history, physical examination, and basic laboratory/imaging investigations. Clinicians should maintain a high index of suspicion for an infectious etiology in patients presenting with a brain lesion in the setting of a recent infection.

## Ethical approval

Not pursued at this time as this is only a clinical image and consent for publication was obtained from the patient. This can be obtained if a requirement for publication in the journal.

## Consent

Written informed consent was obtained from the patient for publication of this case report and accompanying images.

## CRediT authorship contribution statement

**LS, KK** and **JME:** Clinical management of the patient, writing and editing the manuscript. **AW:** Writing, editing the manuscript. **SY:** Interpretation of radiographic imaging, editing the manuscript. **SK:** Interpretation of pathology, editing the manuscript.

## Declaration of interest

The authors report no conflicts of interest.
